# Thinking Animals: A Closed Case or an Open Debate?

**DOI:** 10.3389/fpsyg.2012.00250

**Published:** 2012-07-18

**Authors:** Audrey E. Parrish, Michael J. Beran

**Affiliations:** ^1^Language Research Center, Georgia State UniversityAtlanta, GA, USA

*Animal Thinking: Contemporary Issues in Comparative Cognition* was born out of a meeting on animal thinking through the Ernst Strüngmann Forum. The Forum's main objective is for the continual advancement of scientific knowledge facilitated by bringing together leading experts to address high-priority areas of interest from multiple vantage points. Editors Menzel and Fischer proposed a Forum on comparative cognition to address key topics in the field and to clarify points of contention and highlight progress, both past and potential. This volume is the culmination of the joint effort of an impressive list of 46 leading experts in the field of comparative cognition. Four key areas in animal thinking were addressed in the book in separate sections, including navigation, decision making and planning, communication, and social knowledge, for a total of 18 chapters. There were summary chapters following each section that reviewed the key points and discussed potential areas for new research to further advance these subfields, all of which reflected back on one key topic – animal thinking.

Should the phrase “animals think” be a question or a statement? Should we now conclude that non-human animals have thought processes on par with humans? Comparative psychology has historically straddled the fence between defaulting to over-simplified, non-mental accounts that animals are merely stimulus-response machines to over-qualifying animal behavior with unfounded complex and highly mentalistic interpretations. Editors Menzel and Fischer address the issue of the proper interpretation of animal behavior as it manifests in multiple subfields within cognitive psychology. In doing so, they intentionally left the question mark off of their title, *Animal Thinking*. However, they also correctly noted that in doing this, they were not arguing that similarity in behavior across species means similarity in the mechanisms underlying such behaviors. They instead noted that bringing animals into the fold of cognitive agents, when it is appropriate to do so, helps us better understand human cognition – whereas a non-comparative approach may have blocked progress in understanding our own minds when it isolated human cognition as somehow special and independent of the effects of biological (and psychological) evolution.

Along these same lines, Menzel and Fischer discussed a major point of contention in cognitive psychology: the principle of parsimony, which states that the simpler of two competing theories should be preferred. For animal behavior, this means that the least complex explanation that can account for an observed behavior should be given priority. However, this does not mean that any behavior by a non-human animal must be explained by simple rules and associations. Instead, the principle of parsimony must be applied appropriately as to avoid superfluous interpretations of behavioral phenomena, keeping in mind that this default assumption is not always appropriate and sometimes, a more cognitively enriched interpretation may fit the bill (Smith et al., 2012; also see Shettleworth, 2010).

Some parts of the book merit special mention. We enjoyed the discussion of the “navigation toolbox” of increasing cognitive complexity, which was proposed to include map-less navigation through navigational planning (i.e., eliciting a cognitive map). We found Stevens’ chapter on mechanisms for future decisions to be particularly thought provoking and important to understanding how animals sort through the large number of decisions they are faced with daily. His discussion on bounded rationality nicely outlined the prevalence of mechanisms with simple decision rules over complex computations that yield similar outcomes. He also highlighted that animals (including those of the human variety) are not necessarily optimal decision makers, yet may use simple heuristics that approach optimal outcomes.

Jensen et al. defined social cognition as a suite of cognitive processes that allow an animal to navigate its ever-changing social environment. The authors note the challenge in identifying these mechanisms and the difficulty in distinguishing how multiple species might employ different cognitive processes to culminate in the same functional outcome. These authors also explored whether social and physical cognition are truly distinct in nature, or rather influence and build upon one another, suggesting that while the two are different, they are not separate. This is an idea that has captured our own attention of late, particularly as we have begun examining the cognitive processes underlying social behavior and the study of how physical cognition can be impacted by an animal's social environment. Cheney's discussion of the potential contributions of some cognitive processes (e.g., planning, metacognition) to social behavior such as cooperation is also noteworthy in this regard, and we agreed that this perspective continues to be vitally important. Hampton's reminder that lab researchers should look for natural situations that evoke the cognitive processes studied in the lab is also an important one to remember.

Many of the authors reminded the reader that comparative cognition must remain committed to the “comparative” part of its mission by assessing as many species as can be tested fairly and appropriately given the natural tendencies and behavioral potentials of each given species. Bshary et al. convincingly argued that “species fair” tests are critical to continued development of a truly comparative psychology (e.g., the Transfer Index; Rumbaugh and Pate, 1984), and one must remember that just because multiple species can be presented with the same task does not mean that one is “giving the same test.” Results of poorly conceived tests may over- or under-estimate a species’ true abilities, handicapping or unfairly promoting what those species might show by way of cognitive competence.

We recommend this book to anyone who is interested in “animal thinking” in general, whether you want the question mark to end that phrase or not, because it appropriately addresses the ongoing debate about how to interpret animal behavior. Although this book is probably not introductory or comprehensive enough to serve as an all-encompassing guide to comparative cognition, this is also not its mission, whereas it serves as a valuable resource for graduate students and researchers in comparative psychology interested in the contemporary issues in the field.
